# Real-World Clinical Outcomes in Patients With Multiple Myeloma Administered With Elotuzumab-Based Treatment

**DOI:** 10.7759/cureus.49307

**Published:** 2023-11-23

**Authors:** Taku Kikuchi, Nobuhiro Tsukada, Yuki Oda, Moe Nomura-Yogo, Tomomi Takei, Kota Sato, Mizuki Ogura, Yu Abe, Kenshi Suzuki, Tadao Ishida

**Affiliations:** 1 Department of Hematology, Japanese Red Cross Medical Center, Tokyo, JPN

**Keywords:** anti-cd38 antibodies, triple-class refractory, early treatment lines, disease progression, elotuzumab

## Abstract

Objective

Elotuzumab is used to treat relapsed and/or refractory multiple myeloma (MM). However, the optimal patient selection and sequencing in MM therapy are less clear. Therefore, this retrospective cohort study assessed the clinical outcomes of patients with MM who underwent elotuzumab-based therapy.

Methods

We reviewed the medical records of 85 patients with relapsed/refractory MM who received elotuzumab for the first time. Participants were divided into progressive disease (PD group) and those without PD (non-PD group) at elotuzumab treatment initiation, and each group was analyzed separately. Survival rates were calculated using Kaplan-Meier curves and compared using log-rank tests.

Results

The median follow-up period was 33.6 (range: 0.5-72.0) months. The median progression-free survival (PFS) and overall survival (OS) of PD and non-PD groups at elotuzumab therapy initiation were 5.3 months and not reached (NR), respectively (*P* < 0.0001), and 26.8 months and NR, respectively. Patients with triple-class refractory disease in both groups had worse PFS and OS. Twenty-one patients in the non-PD group received elotuzumab as post-hematopoietic stem cell transplantation, whose PFS and OS were NR (95% CI, 21.4 months-NR) and NR (95% CI, NR-NR), respectively.

Conclusions

Elotuzumab exhibited limited therapeutic efficacy in patients with triple-class refractory MM but better treatment outcomes in situations with adequate disease control and post-transplant treatment.

## Introduction

Elotuzumab is an anti-signaling lymphocytic activation molecule F7 (SLAMF7) monoclonal antibody, which is used to treat relapsed/refractory multiple myeloma (MM) [1]. SLAMF7 is a cellular glycoprotein that is highly expressed by MM, natural killer (NK), and some other immune cells, and it is minimally expressed in normal tissues [2]. Elotuzumab has multiple mechanisms of action against MM cells, including direct NK cell activation, antibody-dependent cytotoxicity via NK cells, antibody-dependent cell phagocytosis via macrophages, and neutralization of soluble SLAMF7 [1-4]. In clinical trials, the combination of elotuzumab with immunomodulatory drugs (IMIDs) and dexamethasone yielded significant improvements in progression-free survival (PFS) and overall survival (OS) when compared with the use of IMID and dexamethasone therapy in patients with relapsed/refractory MM [5-7]. However, the optimal use and timing of elotuzumab-based treatments have not been fully evaluated in the novel agent era. Therefore, we retrospectively evaluated the outcomes in patients with MM treated with elotuzumab.

## Materials and methods

Study design and patients

This retrospective, non-interventional cohort study included data from the electronic medical records of patients with MM treated at the Japanese Red Cross Medical Center (JRCMC). All patients were ≥18 years of age with a documented diagnosis of MM. Patients with relapsed/refractory MM who received elotuzumab for the first time were enrolled in this study. The study was executed in accordance with the principles of the Declaration of Helsinki, approved by the JRCMC Institutional Review Board and conducted after obtaining informed consent from the participants under the opt-out consent principle. None of the participants requested to be excluded from the study.

Definitions

The response to treatment was evaluated according to the International Myeloma Working Group (IMWG) response criteria [8]. The high-risk cytogenetic abnormalities (HRCA) included were del(17p), t(4;14), and t(14;16). Double-class refractory disease was defined as non-responsiveness to proteasome inhibitors (PIs) and IMIDs, whereas triple-class refractory disease was defined as refractoriness to PIs, IMIDs, and anti-CD38 monoclonal antibodies.

OS was defined as the interval between the day of the first elotuzumab dose and the day of death from any cause or last follow-up. PFS was defined as the interval between the first elotuzumab dose and the day of disease progression or death because of any cause. If the patient was switched from elotuzumab to another treatment without progression, their data were censored on the day of initiation of the next treatment to exclude the effect of the next treatment on the treatment outcome. Disease progression was defined according to IMWG response criteria [8].

Statistical analysis

Fisher’s exact tests were used for intergroup comparisons of categorical variables. Data were censored in patients alive or without disease progression at the last follow-up. Survival rates were calculated using the Kaplan-Meier method. Survival rates between groups were compared using the log-rank test. P-values < 0.05 were considered statistically significant. All statistical analyses were performed using EZR software (https://www.jichi.ac.jp/saitama-sct/SaitamaHP.files/statmedEN.html) [9].

## Results

Patients’ characteristics

We extracted the data of 85 patients who received their first dose of elotuzumab at the JRCMC between December 2016 and December 2022.

The characteristics of the 85 patients are shown in Table 1. The median age was 65 years (range: 34-88 years), and 37 patients (43.5%) were female. The median number of prior treatment lines was two (range: 1-8). Thirty-one of the 85 patients had progressive disease at the time of elotuzumab treatment initiation. The remaining patients were switched to elotuzumab-based therapy owing to inadequate response or intolerance of prior treatments. The partner drugs for elotuzumab included lenalidomide (53 patients) and pomalidomide (31 patients). One patient was switched from lenalidomide to pomalidomide, which was continuously administered as a partner to elotuzumab.

**Table 1:** Patient characteristics at the time of elotuzumab initiation (n = 85) Ig: immunoglobulin; IMID, immunomodulatory drug; ISS, international scoring system; LDH, lactate dehydrogenase; MM, multiple myeloma; PI, proteasome inhibitor; ULN, upper limit of normal. ^a^Double-class refractory was defined as refractory to PIs and IMIDs. ^b^Triple-class refractory was defined as refractory to PIs, IMIDs, and daratumumab.

Variable	
Age (years), median (range)	65 (34–88)
Sex, n (%)	
Female	37 (43.5)
Male	48 (56.5)
MM subtype, n (%)	
IgG	58 (68.2)
IgA	14 (16.5)
IgD	2 (2.4)
Bence-Jones	11 (12.9)
ISS stage at diagnosis, n (%)	
Stage I	29 (34.1)
Stage II	34 (40)
Stage III	16 (18.8)
Unknown	6 (7.1)
LDH level, n (%)	
>ULN	16 (18.8)
≤ULN	69 (81.2)
Estimated glomerular filtration rate, n (%)	
≥60 mL/min/1.73m^2^	53 (62.4)
<60 mL/min/1.73m^2^	32 (37.6)
Cytogenetic risk, n (%)	
High-risk	19 (22.4)
del(17p)	10 (11.8)
t(4;14)	13 (15.3)
t(14;16)	1 (1.2)
Standard-risk	27 (31.8)
Unknown	39 (45.9)
Number of prior lines of therapy, median (range)	2 (1–8)
Prior therapy, n (%)	
Proteasome inhibitor	83 (97.6)
Immunomodulatory drugs	82 (96.5)
Anti-CD38 antibody	25 (29.4)
Autologous stem cell transplantation	44 (51.8)
Allogeneic stem cell transplantation	11 (12.9)
Refractory status, n (%)	
PI refractory	45 (52.9)
IMIDs refractory	39 (45.9)
Anti-CD38 antibody refractory	16 (18.8)
Double-class refractory^a^	33 (38.8)
Triple-class refractory^b^	15 (17.6)
Progressive disease at start of elotuzumab, n (%)	31 (36.5)

Overall cohort

The median follow-up period was 33.6 (range: 0.5-72.0) months. The overall response, stringent complete response (sCR), complete response (CR), and very good partial response (VGPR) rates were 63.5% (54 patients), 40.0% (34 patients), 4.7% (four patients), and 8.2% (seven patients), respectively. Hematological adverse events are described in Table 2. Grade 4 neutropenia and thrombocytopenia were reported in 5.9% and 8.2% of patients, respectively.

**Table 2:** Hematologic adverse events

	Grades 3, 4		Grade 4	
Variables (N = 85)	N	%	N	%
Neutropenia	28	33	5	5.9
Lymphocytopenia	48	56.5	11	12.9
Anemia	14	16.5	0	0
Thrombocytopenia	13	15.3	7	8.2

**Figure 1:**
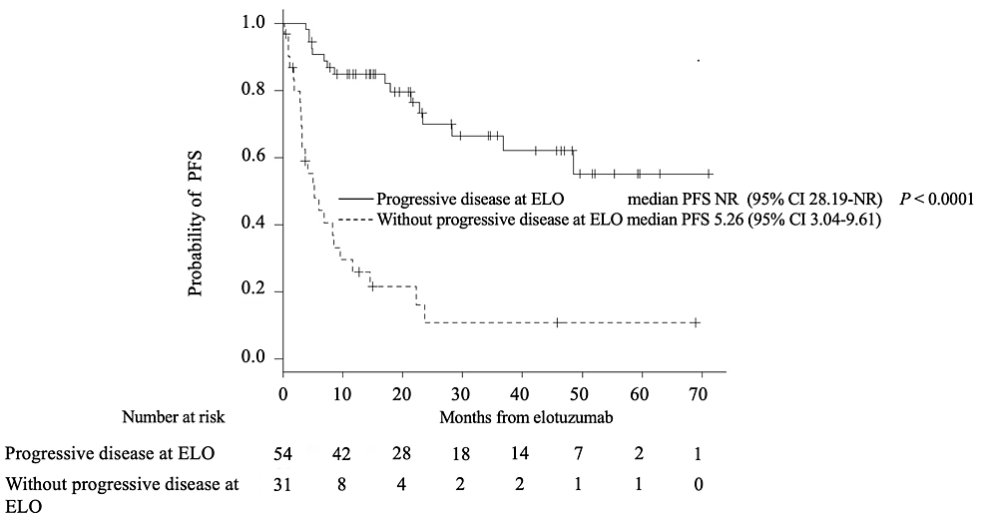
Kaplan–Meier survival curves for progression-free survival from elotuzumab initiation, PFS-stratified progressive disease PFS, progression-free survival; ELO, elotuzumab; NR, not reached

**Figure 2:**
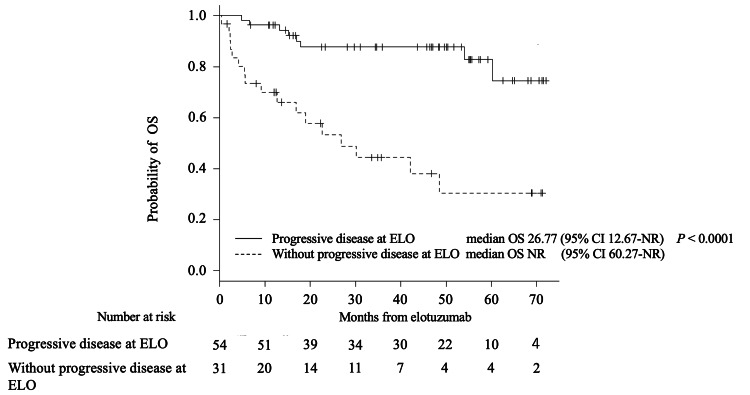
Kaplan–Meier survival curves for overall survival from elotuzumab initiation, OS-stratified progressive disease OS; overall survival, ELO; elotuzumab, NR; not reached

The median PFS and OS of the cohort from the time of elotuzumab administration were 23.7 (95% confidence interval (CI), 17.1 months-not reached (NR)) months and NR, respectively. The one- and two-year OS was 86.9% (95% CI, 77.6-92.5%) and 75.5% (95% CI, 64.2-83.7%), respectively. The median PFS of patients with and without progressive disease at the time of elotuzumab therapy initiation was 5.3 months and NR, respectively (P < 0.0001) (Figure 1). The median OS of patients with and without progressive disease at the time of elotuzumab therapy initiation was 26.8 months and NR, respectively (P < 0.0001) (Figure 2). Thereafter, patients were divided into two groups: those with progressive disease (PD group) at the initiation of elotuzumab treatment and those without (non-PD group), and each group was subjected to separate analyses.

Patients with progressive disease at the initiation of elotuzumab

The median follow-up period was 16.9 (range: 0.5-71.2) months. The median number of prior treatment lines was four (range: 1-8). The overall response rate (ORR) was 16.1%, with an sCR of 6.5% and PR of 9.7%. The median PFS of patients with and without triple-class refractory disease was 3.7 and 8.5 months, respectively (P = 0.0075). The median PFS of patients with ≥3 prior lines and ≤2 prior lines was 3.7 and 16.0 months, respectively (P = 0.027). Moreover, high lactate dehydrogenase (LDH) and the number of prior lines were significantly associated with a shorter PFS, but not renal impairment (eGFR < 60 mL/min/1.73 m3) and HRCAs in the univariate analysis (Table 3). The median OS of patients with and without triple-class refractory disease was 9.2 and 48.5 months, respectively (P = 0.0068). The median OS of patients with and without high LDH was 4.2 and 42.0 months, respectively (P = 0.034). The results of the univariate analysis for OS are shown in Table 3. Although the number of prior lines and HRCAs did not exert an effect on OS, there was a trend towards better OS.

**Table 3:** Factors affecting progression-free survival in univariate analysis in patients with progressive disease CI, confidence interval; eGFR, estimated glomerular filtration rate; NR, not reached ^a^High-risk is defined as postive for del(17p), t(4;14), t(14;16) based on fluorescence in situ hybridization. ^b^Triple-class refractory is defined as refractory to proteasome inhibitors, immunomodulatory drugs, and daratumumab.

			Progression-free survival (PFS)		Overall survival (OS)	
Variables		N	Median (months), (95% CI)	*P* values	Median (months), (95% CI)	*P* values
eGFR at elotuzumab initiation	≥60 ml/min/1.73m^2^	15	5.3 (1.9-8.5)	0.98	30.2 (4.2-NR)	0.38
	<60 ml/min/1.73m^2^	16	5 (2.8-22.3)		42.0 (2.4-NR)	
LDH at initiating of elotuzumab	Normal	24	6.8 (3.1-14.5)	0.0047	42.0 (16.9-NR)	0.034
	High	7	3.0 (0.2-4.2)		4.2 (2.1-NR)	
Cytogenetic risk^a^	Standard-risk	4	NR (1.1-NR)	0.082	12.7 (12.7-NR)	0.13
	High-risk	8	1.9 (0.2-5)		3.5 (0.5-NR)	
Number of prior lines	1-2	21	3.7 (1.8-6.8)	0.027	18.9 (4.2-NR)	0.13
	≥3	10	16.0 (3.0-NR)		48.5 (5.6-NR)	
Anti-CD38 antibody exposed	No	17	9.0 (3.1-23.7)	0.00021	42.0 (12.7-NR)	0.041
	Yes	14	3.2 (0.9-5.3)		18.94 (2.4-NR)	
Triple-class refractory^b^	Not refractory	18	8.5 (3.0-22.3)	0.0075	48.5 (16.9-NR)	0.0068
	Refractory	13	3.7 (0.9-5.3)		9.2 (2.3-NR)	

Patients without progressive disease at the initiation of elotuzumab

The median follow-up period was 46.6 (range: 4.9-72) months. The median number of prior treatment lines was 1.5 (range: 1-6). The ORR was 90.7%, with an sCR of 59.3%, CR of 7.4%, and VGPR of 13.0%. The three-year PFS was 66.5% (95% CI, 48.9-79.2%). The three-year OS was 87.7% (95% CI, 74.5-94.3%). The median PFS of patients with and without triple-class refractory disease was 4.3 months and NR, respectively (P = 0.027). The median PFS of patients with and without anti-CD38 antibody exposure was 17.1 months and NR, respectively (P = 0.0024). According to the univariate analysis, high-risk cytogenetics, high LDH levels, and the number of prior lines were not significantly associated with a shorter PFS (Table 4). The median OS of patients with and without triple-class refractory disease was 5.7 months and NR, respectively (P < 0.0001). The median OS of patients with ≥3 prior lines and ≤2 prior lines was 54 months and NR, respectively (P = 0.0026). Additionally, univariate analysis identified several factors that significantly affected OS, including anti-CD38 antibody exposure. Although HRCAs and high LDH levels had no significant difference in the OS, there was a trend toward better OS (Table 4).

**Table 4:** Factors affecting progression-free survival in univariate analysis in patients without progressive disease CI, confidence interval; eGFR, estimated glomerular filtration rate; NR, not reached ^a^High-risk is defined as postive for del(17p), t(4;14), t(14;16) based on fluorescence in situ hybridization. ^b^Triple-class refractory is defined as refractory to proteasome inhibitors, immunomodulatory drugs, and daratumumab.

			Progression-free survival (PFS)		Overall survival (OS)	
Variables		N	Median (months), (95%CI)	*P* values	Median (months), (95%CI)	*P* values
eGFR at elotuzumab initiation	≥60 ml/min/1.73m^2^	38	NR (23.4-NR)	0.96	NR (60.3-NR)	0.65
	<60 ml/min/1.73m^2^	16	NR (8.6-NR)		NR (17.9-NR)	
LDH at initiating of elotuzumab	Normal	45	NR (23.4-NR)	0.27	NR (60.3-NR)	0.3
	High	9	NR (4.3-NR)		NR (6.6-NR)	
Cytogenetic risk^a^	Standard-risk	23	48.5 (22.8-NR)	0.76	NR (NR-NR)	0.56
	High-risk	11	23.4 (6.9-NR)		NR (17.0-NR)	
Number of prior lines	1-2	14	NR (6.9-NR)	0.19	54 (15.2-NR)	0.0026
	≥3	40	NR (28.2-NR)		NR (NR-NR)	
Anti-CD38 antibody exposed	No	43	NR (36.7-NR)	0.0024	NR (NR-NR)	<0.0001
	Yes	11	17.1 (4.3-NR)		NR (6.6-NR)	
Triple-class refractory^b^	Not refractory	52	NR (28.2-NR)	0.027	NR (NR-NR)	<0.0001
	Refractory	2	4.3 (4.3-NR)		5.7 (4.9-NR)	

Twenty-one patients in the non-PD group received elotuzumab therapy as post-hematopoietic stem cell transplantation (HSCT) therapy, whose PFS was NR (95% CI, 21.4 months-NR) and OS was NR (95% CI, NR-NR). Among 21 patients who received maintenance therapy with elotuzumab after HSCT, seven had a history of allo-HSCT. All seven patients were started on maintenance therapy without graft-versus-host disease (GVHD) at the start of treatment. Two patients were concurrently receiving immunosuppressive agents. The dose of lenalidomide was 5 mg/day in four patients and 10 mg/day in two, and one patient received 20 mg/day in a stepwise increase in dose. No patient experienced a recurrence of GVHD after the initiation of elotuzumab therapy.

## Discussion

This study conducted a retrospective analysis of elotuzumab-based therapy in real-world clinical settings. The analysis revealed significant differences in PFS and OS between the PD and non-PD groups, with better outcomes in the latter. These findings suggest that elotuzumab-based treatment may be more effective in the absence of disease progression. Further analyses were conducted for each group because of substantial differences in treatment outcomes. The median number of prior treatment lines in the PD group was 4, and the ORR was 16.1%. According to the ELOQUENT-2 trial, elotuzumab plus lenalidomide and dexamethasone therapy (ERd) targeting patients with MM who had received a median of two prior treatment lines yielded an ORR of 79% [5]. In the ELOQUENT-3 trial, the ORR of elotuzumab plus pomalidomide and dexamethasone therapy (EPd) in patients with MM who had received a median of three prior treatment lines was 53% [7]. These differences in treatment outcomes may be attributed to the differences in the number of prior treatments between the respective patients included in the current study’s PD group and the ELOQUENT-2 and ELOQUENT-3 trials and the inclusion of 14 patients with exposed anti-CD38 antibody. Consequently, exposure to multiple classes of drugs could have led to the selection of resistant clones, resulting in an inadequate treatment response.

In contrast, a high ORR of 90.7% was achieved in the non-PD group. This outcome surpassed the ORR of 83% observed in the ELOQUENT-1 trial for ERd therapy in patients with newly diagnosed MM [10]. This suggests that elotuzumab-based therapy was particularly effective when administered to patients who had responded well to their prior treatments, indicating the potential of elotuzumab to deepen or sustain the response when used in a setting with good disease control. Indeed, patients who received elotuzumab as post-HSCT therapy showed favorable treatment outcomes. This observation further supports the conjecture mentioned above. In the DSMMXVII trial, the evaluation of elotuzumab maintenance therapy is still underway, and the data have not reached maturity. In the future, the results of this evaluation will provide further clarity regarding the position of elotuzumab as a maintenance therapy [11].

On the other hand, univariate analysis revealed that both PFS and OS outcomes were poor in the patients in the non-PD group who had triple-class refractory disease or exposure to anti-CD38 antibodies. Similarly, even in the PD group, 13 of the 14 patients exposed to anti-CD38 antibodies had triple-class refractory disease with poor treatment outcomes. In the PD group, particularly among patients with triple-class refractory disease, the median OS was 9.2 months, which is consistent with the median OS of 8.6 months in the multiple myeloma: outcomes after therapy failure (MAMMOTH) study [12]. Thus, elotuzumab is considered to have limited therapeutic efficacy in patients with triple-class refractory MM and in patients with anti-CD38 antibody exposure/refractory, making it challenging to achieve significant treatment outcomes. Anti-CD38 antibodies can lead to a decrease in the number of NK cells, and elotuzumab acts by binding to SLAMF7 on myeloma cells, inducing antibody-dependent cellular cytotoxicity (ADCC) through interaction with NK cells via CD16 [2,13]. Furthermore, SLAMF7 is known to be a regulator of NK cell function, and in vitro studies have shown that elotuzumab binding to SLAMF7 on NK cells directly activates them, enhancing their cytotoxic activity against myeloma cells. A significant reduction in the activity of ADCC induced by elotuzumab in the absence of NK cells from peripheral blood mononuclear cells has been reported [2]. Therefore, it is plausible that the decrease in the NK cell population after anti-CD38 antibody exposure may have hindered the induction of effective ADCC by elotuzumab, leading to the lack of efficacy against myeloma. Additionally, the use of anti-CD38 antibodies may have favored the selection of more treatment-resistant clones, contributing to poorer treatment outcomes with elotuzumab. Consequently, these results indicate that elotuzumab should be used prior to anti-CD38 antibody administration. This result is consistent with the findings from previously reported univariate analyses [14].

This study has several limitations. First, this study incorporated a retrospective, single-institution design, however, which reflects homogeneous real-world data. Second, ERd and EPd were analyzed jointly in this study because of the focus on PD and non-PD groups. However, we confirmed that the results of the analysis of ERd and EPd separately also showed almost the same trend as the present results (data not shown).

## Conclusions

In conclusion, this study demonstrated that elotuzumab yielded better treatment outcomes in situations with adequate disease control. Furthermore, the findings suggest that elotuzumab should be used before the administration of anti-CD38 antibodies, exposure to which appeared to reduce the therapeutic efficacy of elotuzumab. These data provide insights into the appropriate use of elotuzumab.
